# Missense-depleted regions in population exomes implicate ras superfamily nucleotide-binding protein alteration in patients with brain malformation

**DOI:** 10.1038/npjgenmed.2016.36

**Published:** 2016-10-05

**Authors:** Xiaoyan Ge, Henry Gong, Kevin Dumas, Jessica Litwin, Joanna J Phillips, Quinten Waisfisz, Marjan M Weiss, Yvonne Hendriks, Kyra E Stuurman, Stanley F Nelson, Wayne W Grody, Hane Lee, Pui-Yan Kwok, Joseph T C Shieh

**Affiliations:** 1 Department of Pediatrics, Division of Medical Genetics, University of California San Francisco, San Francisco, CA, USA; 2 Institute for Human Genetics, University of California San Francisco, San Francisco, CA, USA; 3 Department of Neurology, University of California San Francisco, San Francisco, CA, USA; 4 Department of Pediatrics, University of California San Francisco, San Francisco, CA, USA; 5 Department of Neurologic Surgery, University of California San Francisco, San Francisco, CA, USA; 6 Department of Pathology, University of California San Francisco, San Francisco, CA, USA; 7 Department of Clinical Genetics, VU University Medical Center, Amsterdam, The Netherlands; 8 Departments of Pathology and Laboratory Medicine, Pediatrics, and Human Genetics, Divisions of Medical Genetics and Molecular Diagnostics, University of California Los Angeles, Los Angeles, CA, USA; 9 Department of Pathology and Laboratory Medicine and Department of Human Genetics, University of California Los Angeles, Los Angeles, CA, USA; 10 Department of Pathology and Laboratory Medicine, University of California Los Angeles, Los Angeles, CA, USA; 11 Department of Dermatology, University of California San Francisco, San Francisco, CA, USA; 12 Cardiovascular Research Institute, University of California San Francisco, San Francisco, CA, USA

## Abstract

Genomic sequence interpretation can miss clinically relevant missense variants for several reasons. Rare missense variants are numerous in the exome and difficult to prioritise. Affected genes may also not have existing disease association. To improve variant prioritisation, we leverage population exome data to identify intragenic missense-depleted regions (MDRs) genome-wide that may be important in disease. We then use missense depletion analyses to help prioritise undiagnosed disease exome variants. We demonstrate application of this strategy to identify a novel gene association for human brain malformation. We identified *de novo* missense variants that affect the GDP/GTP-binding site of *ARF1* in three unrelated patients. Corresponding functional analysis suggests ARF1 GDP/GTP-activation is affected by the specific missense mutations associated with heterotopia. These findings expand the genetic pathway underpinning neurologic disease that classically includes *FLNA*. *ARF1* along with *ARFGEF2* add further evidence implicating ARF/GEFs in the brain. Using functional ontology, top MDR-containing genes were highly enriched for nucleotide-binding function, suggesting these may be candidates for human disease. Routine consideration of MDR in the interpretation of exome data for rare diseases may help identify strong genetic factors for many severe conditions, infertility/reduction in reproductive capability, and embryonic conditions contributing to preterm loss.

## Introduction

Genomic sequencing reveals many rare or private heterozygous missense variants per person. These rare variants occur across numerous genes, many of which are not yet associated with any known human disorder, and prioritising variants^1^ in the context of critical genic regions is important for clinical and research applications. Gene alterations responsible for severe human diseases can limit reproductive fitness. Such alterations thus may not be propagated in the population, and therefore coding regions could demonstrate a lack of functional variation in the normal adult population due to intolerance to variation as these variants would have a functional impact.^2,3^ Along this line, taking advantage of the selective pressure on the X-chromosome, where selection pressure would deplete such variation from critical genes, we previously used normal adult X-chromosome data to prioritise potential disease genes and found that genes with missense depletion are more likely to be known human disease genes.^2^ Several illustrative studies have explored measures of variation to examine genic intolerance to variation for predicting and identifying disease genes,^2–5^ but whether such concepts could be applied to specific regions of genes, their proteins, or to specific modes of inheritance^6,7^ has not been fully investigated.

As specific regions of a given gene are more important for function, we rationalise that these can be partially identified as genomic regions that are devoid of missense variation in the normal adult population,^8,9^ and assessment of these regions in an unbiased manner may yield regions more likely to underlie severe rare diseases. Therefore, variant depleted intervals of genes may be intersected with rare missense variants of uncertain significance in exome data of a given individual to facilitate interpretation. When we initially examine the variant pattern from population exome data looking for gene level missense depletion, although the overall missense depletion is modest in disease genes, the greatest difference is seen in genes for dominant or X-linked conditions, which are reported to account for approximately 50% of all diagnoses made for trio clinical exomes.^10–12^ Therefore, a missense depletion strategy could be very useful for the large number of dominant/*de novo* conditions and for identifying new disorders due to unannotated genes.

To apply this hypothesis of MDRs being more likely to cause a human disorder when mutated, here we apply missense depletion to undiagnosed disease exome data where many rare variants of uncertain significance are considered for pathogenicity. By prioritising candidates by missense depletion, we identified a novel gene association of *ARF1* with a human brain malformation. With ARF1 protein, we demonstrate that missense variation near the nucleotide-binding site of ARF1 alters activation and places *ARF1* in the pathway of genes that underlie this birth defect. Importantly, the method described here could be applied more generally for rare variant prioritisation when searching for pathogenic variants for human disorders.

## Results

### Differences in population missense variation are associated with disease gene inheritance pattern

Genic tolerance to variation is useful in evaluating disease gene candidates, especially for candidate genes with loss of function variants. Missense variants, however, are remarkably numerous in the exome, and these variants may be neutral, positive or deleterious depending on the amino acid and the position of the variant within the gene. Such distinction of inferred variant function is essential in predicting the pathogenicity of the variant. However, missense variants are often rare and lack meaningful prior annotation increasing reliance on *in silico* prediction programs such as Polyphen-2 and SIFT. Therefore, alternative methods are needed for evaluating variants and genes for pathogenicity regardless of previous disease annotation or whether a variant has been seen before.

**Figure 1:**
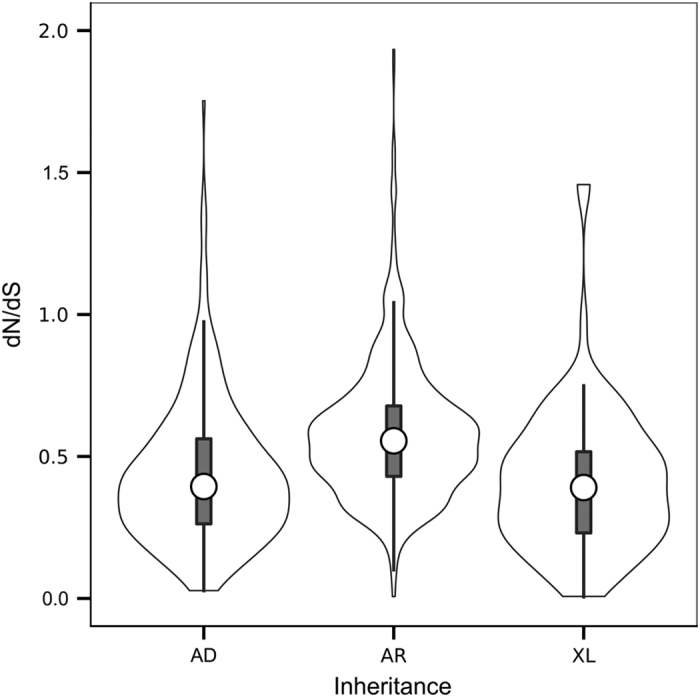
Missense depletion of genes by disease inheritance pattern. Violin plots of d*N*/d*S* for disease genes by inheritance annotation (OMIM and Orphanet). AD, autosomal dominant; AR, autosomal recessive, XL, X-linked. *P*<2.2e−16, Kruskal–Wallis rank sum test.

We and others previously utilised missense-to-synonymous variant ratios in control population exomes (using d*N*/d*S*, which also accounts for sequence composition) to predict disease causing genes^2,3^ but the role of specific gene regions is relatively unexplored.^2^ Several studies have identified disease genes that were predicted by our X-chromosome methods.^13,14^ When we examine all disease genes genome-wide, they show a modest but significant difference in per-gene d*N*/d*S* compared with genes not yet associated with any rare disease (Supplementary Figure 1). These data support the idea of disease genes being more likely to be missense-depleted in the population data. When we analyse known disease genes by disease inheritance pattern, we find that genes causing autosomal dominant or X-linked conditions have significantly lower genic d*N*/d*S* compared with those causing recessive conditions (Figure 1). These results suggest selection against missense variants can be seen in disease genes and seen more significantly in genes where a single-allele deleterious variant is sufficient to lead to a significant phenotype. Given the large portion of exome diagnoses that result from dominant/*de novo* variation,^10–12^ applying missense depletion in prioritising novel candidate genes may be particularly useful even when gene function is unknown or potentially when parental sequence is not available. Importantly, we surmised that the observed missense variant frequency could also vary locally, encompassing not only entire genes but also portions of genes.^2^

### Missense-depleted regions in coding regions of the genome

Hypothesising that genic regions responsible for severe conditions would be depleted of missense variations and could identifying such intragenic regions could lead to finding candidate genes for new dominant conditions in the exome, we analysed all genes in population data from the NHLBI exome sequencing project (ESP) for missense-depleted regions (MDRs).

**Figure 2:**
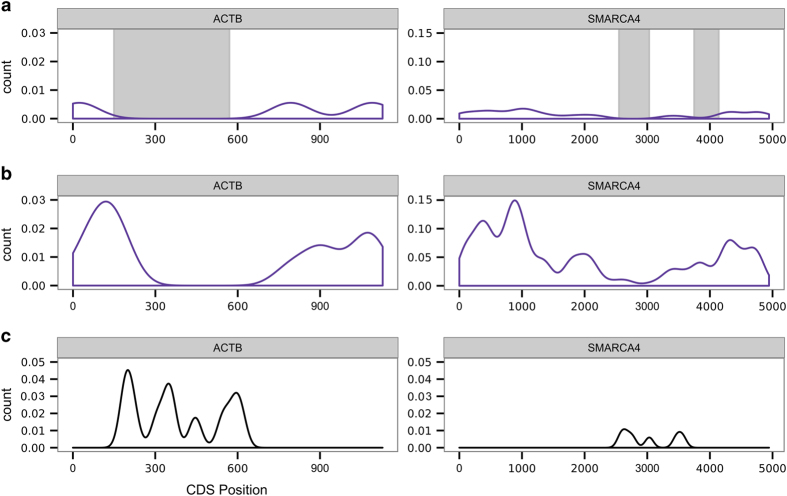
Missense-depleted regions in genes using population exomes. (**a**) Missense variant site density plots along the coding sequence length of *ACTB* or *SMARCA4* from ~6,500 sequenced exomes from ESP. Grey-shaded regions indicate candidate missense-depleted regions (MDRs) that lack missense variants but that have synonymous variants. (**b**) Missense variant plots for *ACTB* and *SMARCA4* using ~60,000 exomes from ExAC data, further delineate MDRs. (**c**) Density plots of pathogenic missense variant sites from ClinVar.

We analysed 18,593 human coding genes and identified genes harbouring missense-depleted regions (defined as at least 300 contiguous base pairs lacking missense variation but harbouring synonymous variants, Methods). One hundred twenty eight genes were identified with regions that completely lacked missense variation. Only 40 of the 128 (31.2%) of these were in genes with known OMIM disease. Plotting missense variation as a function of gene region, distinct segments of coding regions lacked missense variation. These regions can be identified as illustrated in genes such as *ACTB* and *SMARCA4* using the ESP population exome data (Figure 2a), including, for example, crucial nucleotide-binding domains. Further assessment of these Missense-Depleted Regions (MDRs) was performed by considering ExAC data (Exome Aggregation Project) that also includes some of the NHLBI ESP data and increases the number of variants from population exome data (Figure 2b), and MDRs were apparent in *ACTB* and *SMARCA4,* further refining the region. When the locations of known pathogenic mutations for the corresponding syndromes (*ACTB*, associated with Baraitser–Winter and *SMARCA4*, associated with Coffin–Siris),^15–18^ were plotted on the coding sequence, the pathogenic variants overlapped with the MDRs (Figure 2c). These results suggest missense-depleted regions in the genome can be informative for understanding variant effects and for prioritising potential disease variants. MDRs could reveal new candidates for disease in genes/gene regions not yet associated with disease. Indeed, the majority of MDRs are not yet annotated with any human disease. We examined MDR-containing genes using GTEx and 65 of the 128 (51%) were highly expressed in the brain. We provide a list of MDRs identified in the exome and also include genes with overall gene depletion of missense variation (Supplementary Tables 1 and 2). Of the 128 MDR-containing genes, most of the genes are intolerant to loss of function as well (Supplementary Figure 2); however, select genes had loss of function variants (pLI<0.01), suggesting the mechanism of action of deleterious missense variation in select genes is not due to haploinsufficiency. 88% of the cannonical genes had overlap with conserved protein domains.

Given that MDRs identified in the exome could signify important disease gene candidates, we assessed whether genes with MDRs were enriched for any particular molecular functions. Genes with MDRs were highly enriched in nucleotide binding by functional annotation using gene ontology; the top ten enriched terms are shown (Table 1). Thus, nucleotide-binding proteins may be candidates for rare disease and have particular portions that are highly likely to be pathogenic when mutated. We then performed Earthmover's distance (EMD) analysis^19^ (Materials and Methods) as an additional measure to discern differences in distribution of missense and synonymous variants within genes (Supplementary Figure 3 and Supplementary Table 3). We hypothesised that current human exome testing for undiagnosed disease could potentially benefit from testing our observations about patterns of variation. Rare variation within MDRs may need to be prioritised for functional characterisation as disease candidates, and a missense depletion measure could be integrated into exome analyses, potentially increasing the overall diagnostic yield.

### Application to undiagnosed disease exome data

In exome sequencing, a list of filtered variants often includes multiple variants in genes unannotated for disease. We hypothesised that application of missense depletion could be used to prioritise variants in rare undiagnosed disease exome data regardless of knowledge of gene function. Importantly, prioritisation of variants could be performed initially independent of phenotype and considering all rare variants from a proband. To test this, exome sequencing was performed for diagnostic testing for a 9-year-old boy with developmental disability and attention deficit hyperactivity who previously had non-diagnostic genetic tests. Large copy-number variant testing was negative by SNP microarray, and there was no family history of developmental disability. A brain MRI revealed periventricular nodular heterotopia.

**Figure 3:**
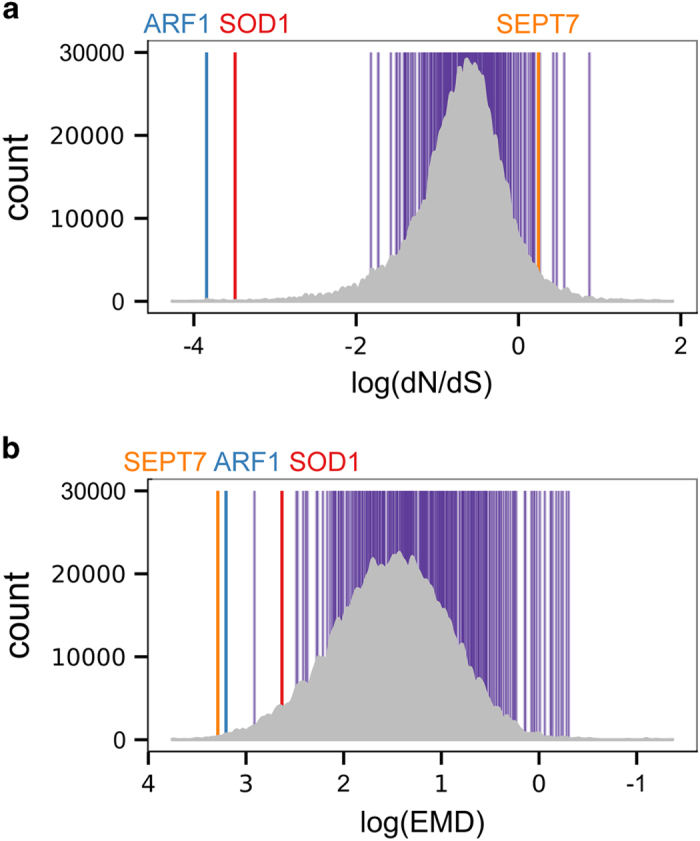
Prioritisation of variant-affected genes from the proband. Comparison of variant-affected genes from the proband (vertical lines) compared with the distribution for all genes in the exome (histogram). (**a**) Evaluation by d*N*/d*S* from population exomes, a lower score indicates higher priority. (**b**) Evaluation using Earthmover's distance, a measure for potential MDR-containing gene. Candidates *ARF1*, *SEPT7* and *SOD1* are highlighted in coloured vertical lines.

Sequencing from the proband yielded a mean coverage of 108× and resulted in 23,323 variants: 22,026 single-nucleotide variants and 1,297 small insertions and deletions (indels). After filtering for amino-acid changing and rare (minor allele frequency <1% in NHLBI ESP population) variants, 480 variants remained. We considered all these variants from the proband exome regardless of prior gene–disease annotation and applied our missense-depletion analysis methods. First, each of the variants was ranked by the corresponding gene overall missense depletion score. Second, each variant-affected gene was also prioritised by its intragenic missense distribution score, EMD, which suggested potential for MDR in the coding region. Either of these methods could be useful in prioritising potential disease-related variants. Interestingly, the distribution of the 480 proband variant-affected genes revealed two genes that were the most missense-depleted: *ARF1* showed the highest priority by missense depletion (Figure 3, Supplementary Table 4), followed by *SOD1*. By intragenic missense distribution score (EMD), *SEPT7* was the highest priority followed by *ARF1* (Figure 3, Supplementary Table 4); *SOD1* ranked 4th by EMD. *ARF1* and *SEPT7* had rare heterozygous variants. *SEPT7,* affected by a missense variant, ranked poorly however by overall missense depletion, at 451st rank, compared to *ARF1* or *SOD1*. Homozygous, mitochondrial and X-linked variants were also considered, but these variants either were found previously in controls or the genes did not rank highly by missense depletion or EMD.

To further examine missense variants in *ARF1* and *SEPT7,* variant locations from the patient exome were examined on variant distribution plots from population exome data (to assess overlap with MDRs). The proband's missense variant in *SEPT7* did not co-localise with the distal MDR in *SEPT7* and was in a region of frequent population missense variation. In contrast, the proband's *ARF1* missense variant (c.103T>C (p.Y35H) NM_001024226) corresponded within the population-data-observed MDR of *ARF1* (Supplementary Table 5). These results indicate that *ARF1* demonstrates missense depletion in population exome data gene-based analysis and also localised missense depletion. Given these findings, the proband missense variant in *ARF1* could be important in disease.

When we added parental exome sequence data (trio analysis including proband), *de novo* and compound heterozygous variants in the proband could also be readily discerned. *ARF1* was among three genes affected by *de novo* variants, and of the *de novo*-affected genes (Supplementary Table 6), *ARF1* was the most outstanding by missense depletion prioritisation using population data (Supplementary Figure 4). The proband's *SOD1* and *SEPT7* variants were inherited from unaffected parent, making these less likely to be pathogenic variants. Analysis of compound heterozygous variant-affected genes (*FLG*, *C10orf92*, *DNAH2* and *ABCA5*) did not reveal other high-priority candidates by missense depletion analyses. The addition of parental data supported the initial findings from proband-only prioritisation by missense depletion. *ARF1* was the top candidate both by proband and by trio analysis. In addition, no variants were observed in the known or possible genes for periventricular heterotopia including *FLNA*, *ARFGEF2* and *ERMARD*, nor were pathogenic variants seen in other known genes implicated for brain malformation syndromes, intellectual disability or ADHD conditions.

### *De novo ARF1* variants in patients with brain malformation

We analysed other population variants reported in this gene to further evaluate ARF1 as a novel disease-associated gene. *ARF1* lacks stop, splice-site or cryptic start variants (predicted high-confidence loss of function) in the NHLBI ESP and in ExAC, however *ARF1* has 8 sites of missense variation in ExAC with one variant observed in 17 individuals in the proximal part of the gene c.16G>A (p.A6T). Examination of the testing lab database also revealed a missense variant that was also reported in ExAC, and this individual did not have the same symptoms as the proband here. We additionally examined both Genome of the Netherlands data, 769 exomes,^20^ and Icelander sequencing data, 2636 genomes,^6^ and did not find missense variation in *ARF1*.

**Figure 4:**
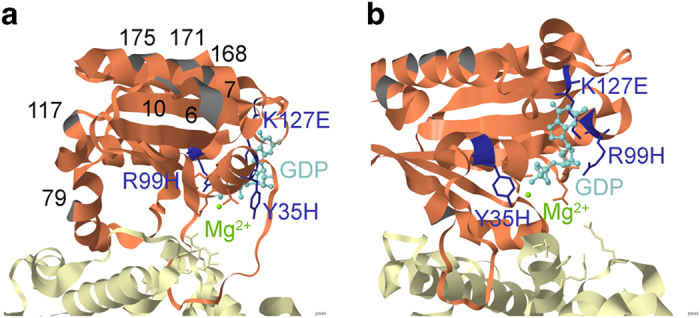
Localisation of variants on ARF1. In blue, location of *de novo* heterozygous c.103T>C (p.Y35H), *de novo* heterozygous variant c.296G>A (p.R99H), and *de novo* heterozygous c.379A>G (p.K127E) from cases. *De novo* missense variants in *ARF1* from brain malformation cases are localised near the GDP-binding site of ARF1 in contrast to the variants found in the ExAC population (dark grey numbers). Colours: ARF1 (orange) and the ARFGEF Sec7 domain, GDP (light blue) and Mg2^+^. (**a**, **b**) are different views of ARF1. Structure from protein database accession 1R8Q.

To understand the potential functional role of the rare population variants in *ARF1* and from the neurologic phenotype, we examined all these variants on the ARF1 crystal structure (Figure 4a,b). ARF1 is a RAS-superfamily GDP nucleotide-binding protein that leads to GDP/GTP conversion and GTP-activation of ARF1 with the help of ARFGEFs. Interestingly, our undiagnosed patient's c.103T>C (p.Y35H) variant is located adjacent to the GDP-binding site of ARF1 and Y35 is predicted by ligand prediction method LPC/CSU (Materials and Methods) to interact with GDP-binding site residues. In contrast, population-detected rare missense variants were localised away from nucleotide-binding residues.

In addition to the patient with p.Y35H, a second unrelated individual with periventricular heterotopia with a *de novo*, rare missense variant in *ARF1*, c.379A>G (p.K127E) was also found. This variant affects an amino-acid residue in the predicted ARF1 GDP-nucleotide binding site by LPC/CSU analysis (Figure 4 and Supplementary Table 7). In addition, the affected residue p.K127 is known to be important in functional studies of ARF1 in other systems.^21,22^ A third patient had a *de novo* missense variant in ARF1 c.296G>A (p.R99H) and had brain malformation with delayed myelination in childhood and significant cerebral underdevelopment 12 years later, and p.R99 appears to contact two of the GDP-binding residues of ARF1 by LPC/CSU analysis (Figure 3 and Supplementary Table 7). The three ARF1 missense variants were in MDR (Supplementary Figure 5). The initial patient on exam had a head circumference of 60th centile, weight of 70th centile, height of 60th centile. He was born at term. He had a history of otitis media and developmental delay especially in speech, he did not have a history of seizure, although he had an EEG in the past. The MRI brain showed periventricular heterotopia and diminished white matter. The second patient had delays, particularly in speech and seizures. When she was seen at 15 years she had a head circumference was 5th centile, the weight was 20th centile, height 3th centile and she had spasticity and had regressed in language abilities. The MRI showed delay in myelination and cortical thinning and vermis atrophy. The third individual had limited information available but had seizures and periventricular heterotopia.

**Figure 5:**
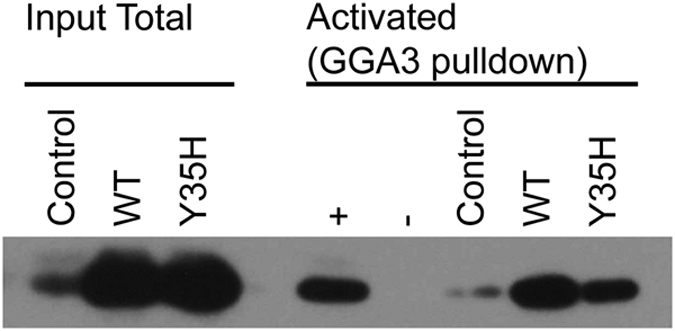
Variant in ARF1 alters nucleotide activation. ARF1 nucleotide activation of wildtype (WT) or p.Y35H ARF1-transfected 293T cells. 293T cells basal ARF1 expression (lane 1), and wildtype and p.Y35H transfection demonstrate similar ARF1 expression (lanes 2 and 3). Blank (lane 4) Pulldown for GTP-activated ARF1 (lanes 5–9): Basal activated ARF1 (lane 7). Compared to activated ARF1 levels in WT (lane 8), p.Y35H variant demonstrates reduced GTP activation (lane 9). Lanes 1–3 represent the protein expression for the assay. Lanes 5–6 represent the positive and negative controls (GTP-gamma-S and GDP, respectively). Result is representative for three independent transfection experiments with pulldown and western blotting.

These results further emphasise the importance of variant prioritisation based on the location of intragenic missense variants, nucleotide-binding proteins and missense depletion regions. Given that the variant p.Y35H in *ARF1* was untested but would be predicted to affect GDP/GTP activation and lies in the G4 loop adjacent to the *ARF1* switch region, we directly tested its effect on ARF1 protein activity. We used site-directed mutagenesis to introduce the variant for p.Y35H into ARF1 cDNA and verified this by sequencing prior to transfection. Wildtype and p.Y35H-altered ARF1 were expressed in 293T cells at similar levels. As opposed to wildtype, however, p.Y35H ARF1 was markedly reduced in nucleotide activation (Figure 5), thus verifying that the missense variation predicted to affect the GDP site was deleterious.

Taken together, these results suggest decreased activation of *ARF1* could be an important factor in disease in the brain. Mutations in *ARF1* have not been associated with disease prior to this report. Interestingly, *ARF1* is likely in a molecular pathway with known genes for periventricular heterotopia including *FLNA* and *ARFGEF2*, the gene encoding the ARF-activator protein BIG2^23^. These results indicate that exome variants may be prioritised using missense depletion methods from population exome data independent of knowledge of a gene or gene region's function. This information combined with functional data can yield new insights into the role of rare variants in human disease.

## Discussion

Medical exomes have tremendous diagnostic potential, but key genetic contributors to disease are elusive in more than half of patients. A significant challenge is interpreting the many rare variants that lack gene/disease annotation. While further sequencing will continue help to identify more variants in the human exome, we demonstrate here a methodology using population missense depletion data to help interpret exome results and prioritise missense variants more likely to have a functional consequence. We demonstrate how variants from exome testing can be examined independent of phenotype. Using these methods, we discover *de novo ARF1* missense variants that affect the protein proximal to its nucleotide-binding site. Moreover, our results suggest decreased ARF1 activity is seen with the brain malformation, and this supports a new potential gene association.

As a known Ras-superfamily GDP/GTP exchange protein, ARF1 is a class I Arf important in membrane traffic^24^ and in mediating COPI vesicle formation. Found in neuronal processes, ARF1 also affects cellular cytoskeletal regulation,^25–27^ as does the X-chromosome encoded FLNA. The identification of *ARF1* variants here suggests an effect in brain development and further extends the *FLNA*-associated pathway in periventricular heterotopia.^28–30^ Interestingly, mutations in *ARFGEF2* encoding BIG2 has been associated with recessive periventricular heterotopia, and these predict loss of ARF-activation in the disorder. On the basis of our functional data, our results also support haploinsufficiency of the ARF pathway leading to brain malformation in these patients. Levels of ARF1 activity may be important to examine further since complete loss of function is embryonic lethal.^31^ The 1q42 genomic region, where *ARF1* resides, has also been implicated in brain development and white matter abnormalities, and our studies add *ARF1* to the candidates that could influence the brain.^32^ Future attention to ARF1 and other GTPases could assist in our understanding neuronal pathogenesis and other intellectual disabilities. Furthermore, our results indicate that nucleotide-binding protein genes are enriched in missense-depleted regions in population exomes. Interestingly, GTPase signalling and nucleoside metabolism were identified in Uddin *et al.*^8^ as pathways important when studying autism spectrum disorder. Therefore, further attention to these proteins is suggested when analysing exome variants in significant neurophenotypic conditions.

The results here show how a prioritisation strategy with MDR can be used in analysing exome data. These strategies could augment variant evaluation methods.^1^ Missense variants represent the largest category of variant-type from exome data and more missense variants will also result from further exome and genome sequencing, and phenotype-agnostic prioritisation of these can assist in data interpretation of probands and trios. Substantial attention can still be given to known gene–phenotype associations, but we hope to augment the identification of novel gene–disease association using the methods described. As aetiologic *de novo* mutations have been frequent in exomes,^5,10,33^ we expect that MDRs will further increase exome yield by annotating portions of genes that are likely to be particularly deleterious when mutated. Application to *de novo *dominant and X-linked conditions may yield new candidate disease genes; however, the application in recessive disease genes will be limited. Regions that are missense-depleted in an increasing sequenced population may represent critical genomic regions that have significant medical importance. Even in known disease genes, they may identify new phenotypes associated with variants in these regions. Furthermore, genetic elements that are missense-depleted could underlie multiple types of conditions with decreased reproductive fitness and therefore could have even broader clinical utility.

## Materials and methods

### MDR analysis

Missense-depleted regions (MDRs) were initially identified by analysing all RefSeq coding sequences (CDS) for regions lacking missense variation in population data. Population exome variant data were obtained from the NHLBI GO ESP (Exome Sequencing Project ESP6500SI-V2-SSA137 http://evs.gs.washington.edu/EVS/; accessed on 25 September 2014) and the ExAC data (Exome Aggregation Consortium r0.3 ftp://ftp.broadinstitute.org/pub/ExAC_release/; accessed on 02 February 2015). The ESP data set includes variants from 6503 exomes. The ExAC data set includes variants from 60,706 exomes (including some from the ESP). ESP exomes are derived from adults (except for some from cystic fibrosis individuals), while the ExAC data set excludes data from patients with severe paediatric diseases. All RefSeq CDS (32,848 transcripts) were analysed for overall and regional missense depletion by quantifying the missense substitution rate and synonymous substitution rate within the population. For identifying MDRs with the most depletion in missense variants, we scanned for coding regions that lack missense variation using a minimum window size of 300 base pairs length, while requiring the presence of at least 10 synonymous variants at a frequency of one for every 10-codon group. The size is a strict criterion for MDRs; we performed analysis for smaller MDRs. With small regions, these could be subject to a lack of missense variants simply due to low numbers of variants, and size resolution should improve with further exome data. We have added Supplementary Figure 6 depicting the number of candidate regions identified with different MDR size criteria.

Missense depletion was further assessed by screening all CDS, assessing the differences in missense and synonymous variant distributions along each CDS length for potential regional missense depletion. CNVs were not analysed due to limited data. The Earthmover's distance (EMD) was used here as a regional measure of distance between two probability distributions based on a Mallows distance with equal masses.^19^ This distance represents the energetic cost of transforming one distribution into another; here we used the distributions of missense and synonymous variants across each CDS. The EMD was calculated using the emdist package for R (http://cran.rproject.org/web/packages/emdist/index.html). Genic d*N*/d*S* was also calculated for all transcripts using variants using the Nei and Gojobori method.^34^ This method tests whether mutations will result in synonymous or missense substitutions for each nucleotide in the coding sequence to determine the number of potential sites in a coding sequence on a per-codon basis. The total *N* sites for the sequence is the sum of the *N* sites for each codon, and similarly the total *S* sites is the sum of all *S* sites in the sequence. These are combined with the actual observed *N *and *S* sites in the population variant data for d*N*/d*S*. EMD and d*N*/d*S* genome-wide statistics from population data were then used to evaluate all variants from exome data.

### Functional analyses

Genes with MDRs were analysed for functional categorisation using the DAVID Functional Annotation tools (https://david.ncifcrf.gov/). We tested genes for enrichment (default thresholds: EASE 0.1, count 2) in any gene ontology term and applied Bonferroni multiple testing correction.

### Gene disease annotation and inheritance patterns

Gene annotation for human diseases were determined based on annotated OMIM disease phenotypes and phenotypes from Orphanet (http://omim.org/, http://www.orphadata.org). Disease annotations shared between the two databases (*n*=2485) were used to minimise differences in annotation by source. We examined phenotypes annotated in both databases or annotated by either source and we found similar results using both these methods for missense depletion analyses. Inheritance patterns were captured from both OMIM and Orphanet, and missense and synonymous substitution rates were compared between genes causing diseases of different inheritance pattern. For the 10% of disease genes with multiple disease inheritance types, inclusion or exclusion of these genes into different inheritance categories also yielded comparable results in missense depletion analyses.

### Exome data analysis in undiagnosed patients

Exome sequencing was performed using blood samples from individuals after informed consent. Studies were performed under The UCSF Committee for Human Subjects Research. Genomic DNA was extracted and sequencing performed using Illumina HiSeq2500 following exon capture. Following QC and duplicate marking, variants were aligned to reference and variants called using GATK. Variants were filtered for amino-acid alteration and then for <1% minor allele frequency based on ESP, NIEHS EGP and the Hapmap project. For proband analysis, all variants meeting these criteria were examined using missense depletion methods regardless of if the variant/gene had previous annotation. Following addition of parental exome data, trio analysis was also performed and *de novo*, compound heterozygous and inherited heterozygous variants could be further annotated. Variants were also examined for previous annotation in HGMD and ClinVar.

### Structural analysis of *ARF1* variants

For protein structure analyses, amino-acid differences from ESP/ExAC population and patient variants were visualised on protein crystal structures using Jmol Viewer using RCSB PDB (http://www.rcsb.org/). The human ARF1 structure 1R8Q, complexed with sec7 and GDP, was used to visualise variant positions in structure. Examination of variants in alternative human structure 1HUR revealed similar results. Amino-acid potential interactions with GDP-binding site residues were analysed using Ligand-protein contacts and contacts of structural units (LPC/CSU; http://ligin.weizmann.ac.il/cgi-bin/lpccsu/LpcCsu.cgi).^35^ Amino acid to ligand contact prediction for variant sites is shown in Supplementary Materials.

SIFT, Provean, and Polyphen2 scores for the variants are shown in Supplementary Table 8. GeneMatcher was used to help study this gene.^36^

### Analysis of ARF1 activation

The effect of p.Y35H on ARF1 was tested by introducing the variant by site-directed mutagenesis into the *ARF1* cDNA plasmid (BC011358). Both wildtype and mutant plasmids were transfected into cultured 293T cells (UCSF cell culture facility) using Lipofectamine and total expression assessed by western blotting for ARF1 following protein quantification. For assessment of activated ARF1, GST-GGA3 (the binding partner that binds GDP/GTP-bound ARF1) pulldown was performed on lysates prior to Western blotting. Processing of samples was performed at 4 degrees and with incubation with GST-GGA3-PBD for 1 h (ThermoFisher, Waltham, MA, USA). Positive control for activation was GTP-gamma-S, negative was GDP per manufacturer's instruction. Western blotting for ARF1 was performed using rabbit anti-ARF1 antibody (catalogue number 16121, ThermoFisher). The experiment was performed with three biological replicates.

### Statistical analysis

We performed statistical tests using R (http://www.r-project.org). The two-sample Wilcoxon test or Kruskal–Wallis Rank Sum test for more categories was used for missense to synonymous ratio comparisons between categories, as variance between groups was not equal. Bonferroni multiple testing correction was utilised in functional gene enrichment testing. Histograms and plots were made with R graphics (http://www.r-project.org) and the ggplot2 package for R (http://ggplot2.org/).

### Code availability

All code is available on request by contacting the corresponding author. Variant data are being deposited at NCBI.

## Figures and Tables

**Table 1:** Enrichment of MDR-containing genes for gene ontology terms

*Category*	*Term*^a^	P-*value*	*Fold*	*Bonferroni*
MF	GO:0000166 nucleotide binding	1.05e−07	2.17	1.13e−04
MF	GO:0032553 ribonucleotide binding	1.35e−07	2.34	1.28e−04
MF	GO:0032555 purine ribonucleotide binding	1.35e−07	2.34	1.28e−04
CC	GO:0043005 neuron projection	4.14e−07	5.40	1.38e−04
MF	GO:0017076 purine nucleotide binding	2.26e−07	2.24	3.87e−04
BP	GO:0030001 metal ion transport	1.16e−06	4.40	6.81e−04
MF	GO:0032559 adenyl ribonucleotide binding	1.76e−06	2.40	9.79e−04
BP	GO:0006812 cation transport	5.33e−07	3.92	1.54e−03
MF	GO:0046873 metal ion transmembrane transporter activity	2.74e−06	4.75	1.91e−03
MF	GO:0005524 ATP binding	3.52e−06	2.36	2.18e−03

Abbreviations: BP, biological process; CC, cell compartment; GO, gene ontology; MF, molecular function.

a Top 10 highest enriched terms are listed.
