# Supplementary material for: Missense-depleted regions in population exomes implicate ras superfamily nucleotide-binding protein alteration in patients with brain malformation
Source: NPJ Genom Med. 2016 Oct 5;1:16036–. doi: 10.1038/npjgenmed.2016.36 (PMC5576364; doi:10.1038/npjgenmed.2016.36)
Supplement: Supplementary Figures [file npjgenmed201636-s1.pdf]

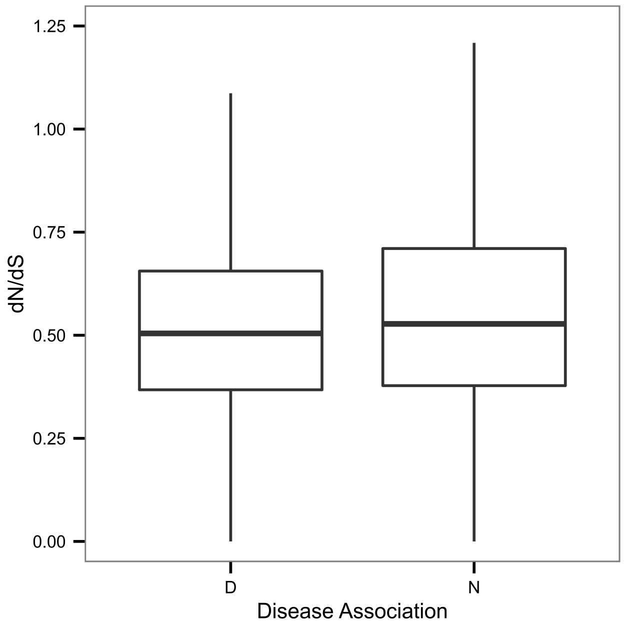

Supplemental Figure 1 Comparison of genic dN/dS ratio by disease association. Disease-associated genes (D) demonstrate modestly lower dN/dS than non-disease-associated genes (N). All genes with disease-association annotation as assessed in both OMIM and Orphanet exome-wide are shown. p = 1.625e-07, Wilcoxon Rank Sum test.

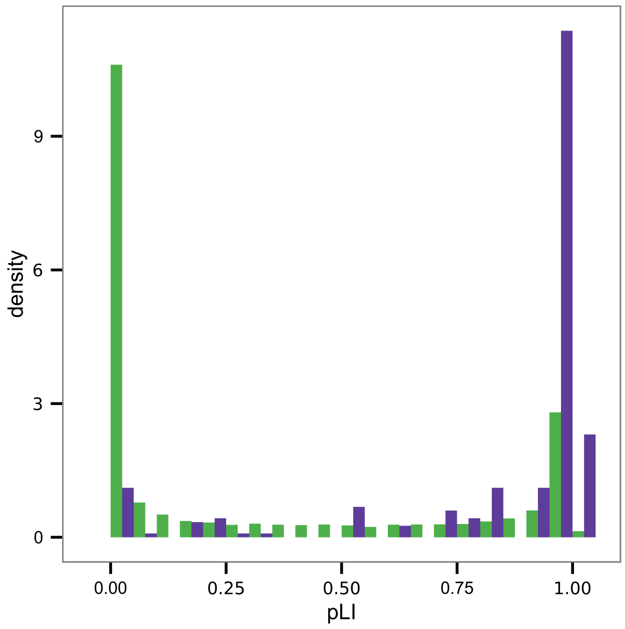

Supplemental Figure 2 MDR-containing genes and pLI.
The 128 MDR-containing genes (purple) demonstrate higher pLI than all 18225 genes (green). Plotted genes' ExAC pLI data. $p < 2.2e-16$, two-sample t-test.

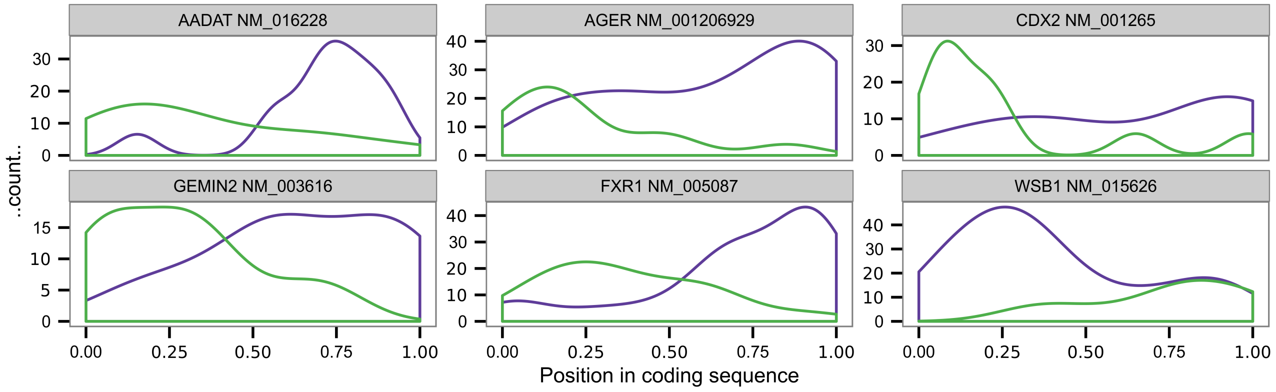

Supplemental Figure 3 The Earthmover's Distance is a Measure of Difference of Missense and Synonymous Variation Distribution.

Missense (purple) and synonymous (green) variation site density plots are shown for genes with high Earthmover's Distance scores and at least ten missense and ten synonymous variants in ESP.

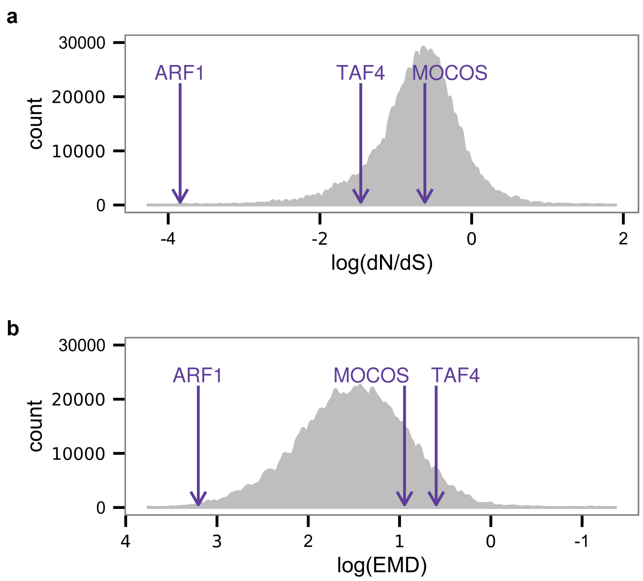

Supplemental Figure 4 Comparison of *de novo* variant-affected genes from undiagnosed disease exome.

Variant-affected genes (arrows) compared to distribution of all genes (histogram). (a) Evaluation of candidates by dN/dS compared to population exomes. (b) Evaluation using Earthmover's Distance.

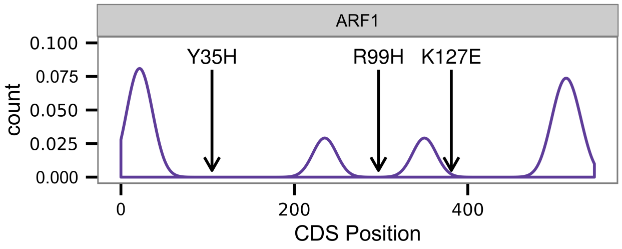

Supplemental Figure 5 Distribution of missense variants in ARF1.
The *de novo* variants (arrows) compared to distribution of missense variants in ExAC (purple).

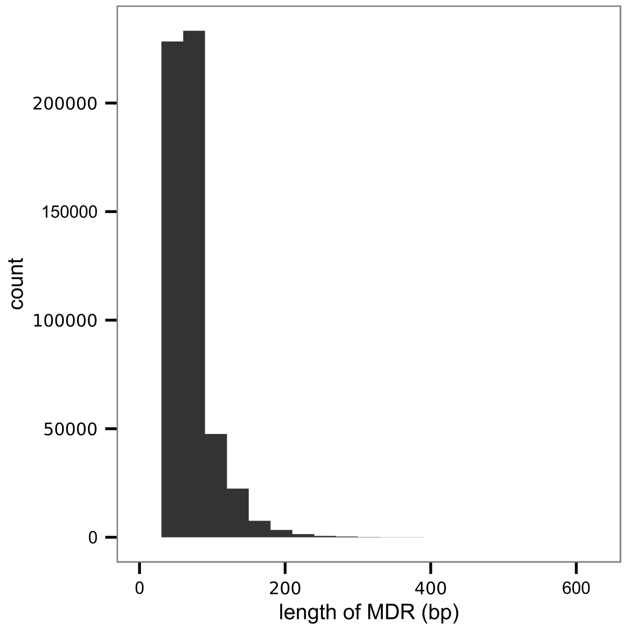

Supplemental Figure 6 Distribution of candidate regions by region size.
The MDR size criterion was varied in 30bp bins. The distribution demonstrates that 300bp is a strict cutoff.
